# Alterations in the Plasma Protein Expression Pattern in Congenital Analbuminemia—A Systematic Review

**DOI:** 10.3390/biom13030407

**Published:** 2023-02-22

**Authors:** Bailey M. Foster, Afsoun Abdollahi, Gregory C. Henderson

**Affiliations:** Department of Nutrition Science, Purdue University, West Lafayette, IN 47907, USA

**Keywords:** hypoalbuminemia, rare disease, blood

## Abstract

Albumin is a highly abundant plasma protein with multiple functions, including the balance of fluid between body compartments and fatty acid trafficking. Humans with congenital analbuminemia (CAA) do not express albumin due to homozygosity for albumin gene mutation. Lessons about physiological control could be learned from CAA. Remarkably, these patients exhibit an apparently normal lifespan, without substantial impairments in physical functionality. There was speculation that tolerance to albumin deficiency would be characterized by significant upregulation of other plasma proteins to compensate for analbuminemia. It is unknown but possible that changes in plasma protein expression observed in CAA are required for the well-documented survival and general wellness. A systematic review of published case reports was performed to assess plasma protein pattern remodeling in CAA patients who were free of other illnesses that would confound interpretation. From a literature search in Pubmed, Scopus, and Purdue Libraries (updated October 2022), concentration of individual plasma proteins and protein classes were assessed. Total plasma protein concentration was below the reference range in the vast majority of CAA patients in the analysis, as upregulation of other proteins was not sufficient to prevent the decline of total plasma protein when albumin was absent. Nonetheless, an impressive level of evidence in the literature indicated upregulated plasma levels of multiple globulin classes and various specific proteins which may have metabolic functions in common with albumin. The potential role of this altered plasma protein expression pattern in CAA is discussed, and the findings may have implications for other populations with hypoalbuminemia.

## 1. Introduction

Serum albumin is the most abundant protein in circulation in humans. Important lessons about the role of albumin in metabolism and physiology can be learned from studies of albumin-deficient humans and laboratory animals. Congenital analbuminemia (CAA) is a rare condition in humans that results in the complete absence or nearly complete absence of albumin expression. This apparently rare condition results from an individual being homozygous for albumin gene mutation. While there have been less than 100 reported cases of CAA [1], this apparently low incidence of 1 per 1,000,000 individuals [1] may be a significant underestimation of cases because of the potential for many undiscovered cases. As the phenotype in CAA patients is not extreme [1], it may go undiscovered in certain individuals who do not participate in preventative medical care that includes biochemical assessment of plasma. Despite the expected roles of albumin in metabolism, CAA patients can live well into adulthood, often without any severe detrimental impacts and without any obvious impact on lifespan [1]. When diagnosis of CAA in a patient is made, the diagnosis would potentially be simply by chance when the patient undergoes routine bloodwork for preventative care or treatment of other health conditions [2,3]. In other instances, as mild edema is the primary symptom of CAA, an investigation to find the root cause of edema may lead to the discovery of CAA [4,5].

It has been speculated that the ability to survive with CAA while exhibiting minimal or no symptoms is dependent upon the patient’s degree of compensation; some investigators have suggested that the expression level of other plasma proteins increases substantially in CAA in order to compensate for absence of albumin [1,6,7]. It is reasoned that elevating the expression of other proteins would minimize the reduction in plasma oncotic pressure that is theoretically expected in CAA. Hypothetically, if this compensation were nearly complete, then the total protein concentration in CAA patients would be normal, even despite the absence of albumin. While in individual case reports plasma protein concentrations have been reported, we are unaware of a systematic review to address the degree to which the plasma protein profile and total protein concentration are modified in CAA patients. This information is important to establish whether compensation in the plasma protein expression levels adequately overcomes the effects of albumin deficiency upon overall plasma protein abundance. 

Some may be surprised that CAA patients can survive to an advanced age, and it is possible that remodeling of the plasma proteome explains this observation. It is also possible that any detrimental effects of albumin deficiency may be balanced by some metabolically protective changes in lipid metabolism. For example, albumin knockout mice exhibit lower plasma free fatty acid (FFA) abundance than seen in wild-type mice [8], and this reduction could have some positive effects, as higher plasma FFA concentration is believed to lead to various health complications [8,9]. However, it is also noteworthy that the effects of complete albumin deficiency might include elevation of low density lipoprotein cholesterol (LDL-C) above the reference range; it is difficult to assess the magnitude of this effect, as typically in case reports patients are not directly compared to appropriate control subjects, and they are instead compared to reference ranges for each analyte. People without CAA often exhibit elevated serum lipids as well, when compared to reference ranges. For example, over half of American adults are classified as being afflicted with dyslipidemia [10] despite wide access to lipid-lowering medications, and this propensity toward elevated LDL and total cholesterol would be expected around the world in social circumstances that provide an abundance of food access. Nonetheless, it might be that CAA (or specifically the reduced total protein concentration in plasma) could lead to further worsening of LDL cholesterol and apolipoprotein B (apoB) expression. Thus, some lipids (e.g., FFA) may be reduced in albumin deficiency while others (e.g., LDL-C) may be elevated. The information compiled about plasma proteins could shed light upon the complex physiology that occurs in CAA patients. Therefore, we performed a systematic review of published CAA case reports to assess the results for plasma protein abundances. 

## 2. Methods

The study followed the preferred reporting items for systematic reviews and meta-analyses (PRISMA), along with PICOS (population, intervention, comparison, outcome, and setting) in Table 1 to define the research question. 

### 2.1. Article Selection

**Figure 1:**
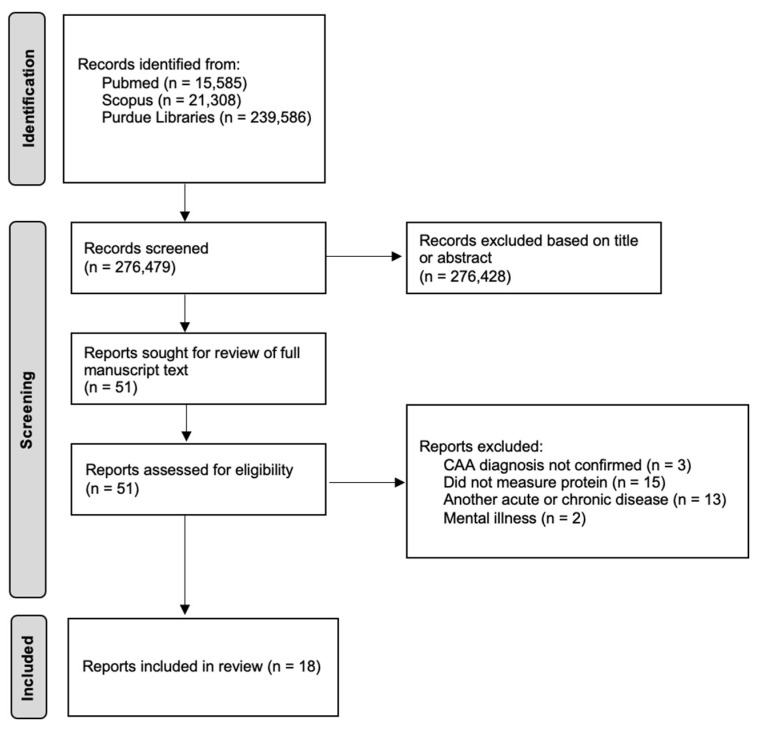
**Flow diagram for systematic review.** Articles were identified and screened based on titles and abstracts from databases. Next, the full text of selected reports were assessed for meeting the inclusion criteria for the systematic review. A paper was selected if at least one patient described in the report met the inclusion criteria. A total of 18 papers were identified, with 23 patients that met the inclusion criteria.

The combined searches on PubMed, Scopus, and the Purdue University Libraries gave a total of 276,479 results (PubMed: 15,585, Scopus: 21,308, Purdue Libraries: 239,586). The systematic review of the literature occurred from 22 September 2021 to 29 October 2021. The literature was last searched on 19 October 2022 prior to submission. The search syntax included “CAA” or “analbuminemia” or “albumin deficiency”. Search filtering for the classification “human” was performed in PubMed and Purdue Libraries, to focus the search toward relevant articles. Only articles written in English were considered. A flow diagram for article screening and selection is shown in Figure 1. Titles were reviewed first, and then abstracts of potentially relevant papers were assessed, leading to identification of 51 potentially relevant papers; for example, this entailed exclusion of papers that were not of human subjects or were clearly studying a topic other than CAA. Next, papers from this group of 51 publications were selected for further review if CAA was confirmed through a mutational analysis; we also selected papers for further assessment if we could reasonably infer a diagnosis of CAA based on a biochemical measurement of serum albumin concentration without the presence of other causes of low serum albumin such as kidney and gut diseases; 48 papers were identified in this step. Next, papers were removed if they did not measure total plasma protein concentration, protein classes, or individual protein concentration; after this step, 33 papers remained. From these 33 remaining articles, additional articles were excluded from our search if patients were diagnosed with another acute or chronic disease or illness except for obesity, hypercholesterolemia, and edema; following this step, 20 papers remained. Two articles were also excluded due to apparent mental illness, reducing the number of final papers to 18. Various illnesses led to exclusion of papers, with no apparent theme other than the observation that 4 of the excluded papers were cases of pulmonary dysfunction that was in patients who had been born very prematurely. Other examples of medical conditions in excluded papers were individual cases of spinal cord injury, epilepsy, and rheumatoid arthritis. Out of the 15 excluded papers, in 7 papers it was reported that the CAA patient was a child of consanguineous parents. A total of 18 articles were selected through this final exclusion process, and from these papers, 23 patients met the inclusion criteria described above and thus were included in the analysis. Individual protein measurements were reported for all 23 patients, and total protein measurement for 18 patients. Each of the articles was selected based on the agreement of all authors. The articles and their protein fold changes are listed in Table 2. 

### 2.2. Protein Selection

In the 18 articles selected for this systematic review, data for a total of 23 patients were included in the systematic review. We counted the number of male and female patients for which each of the proteins was reported. A protein was included in the analysis if it was reported in at least 3 patients of the same sex that met the review’s inclusion criteria. α-1 antitrypsin, ceruloplasmin, haptoglobin, transferrin, immunoglobulin M (IgM), IgA, and IgG, fibrinogen, apoB, and transthyretin were selected based upon this approach, as well as protein classes α-1 globulin, α-2 globulin, β globulin, and γ globulin. After the proteins were selected by counts, it was determined for each patient if a protein was increased (higher than reference range), decreased (lower than reference range), or there was no change (within the reference range). 

## 3. Results

**Figure 2:**
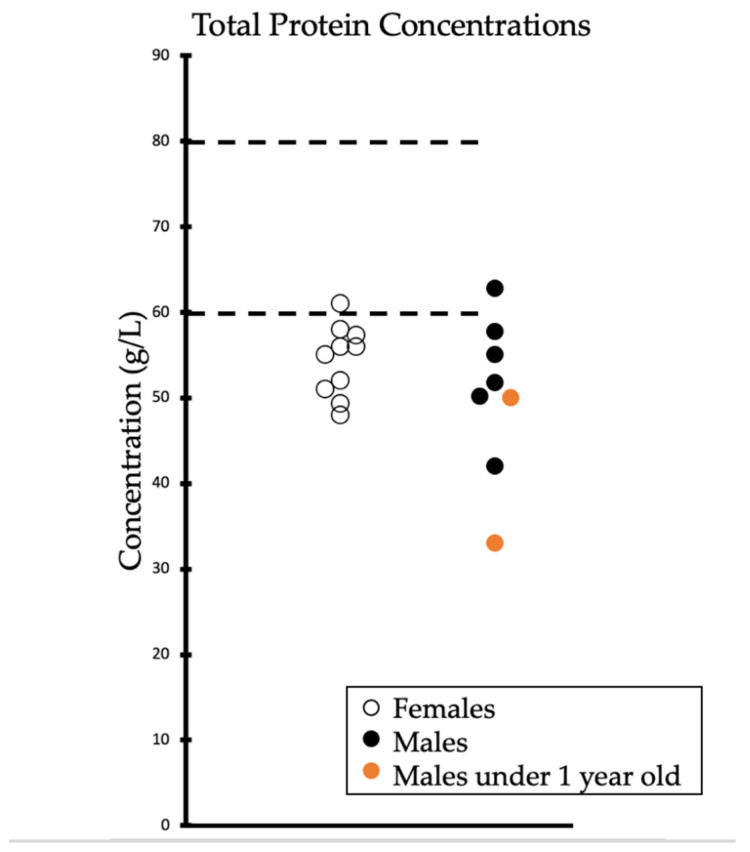
**Total plasma protein concentration in CAA patients.** Each data point represents the total plasma protein concentration measured in an individual with CAA. The filled circle and the unfilled circle represent males and females, respectively. The typical reference range for individuals older than 1 year of age are provided by the dashed lines (60–80 g/L). The orange data points represent the two patients who were younger than one year of age; both of these patients were male.

In the articles included in the analysis, the lower end of the total protein reference range varied between reports from 56–66 g/L, while the upper end of the reference range varied from 75–87 g/L. The clinical reference range commonly used for serum albumin is approximately 60–80 g/L [24], such as 63–79 [25] or 63–82 [26], and these ranges primarily apply to patients older than one year of age. All patients exhibited plasma protein concentration near or below the lower end of this typical reference range (Figure 2). 

To reiterate, proteins or protein classes were included in our literature assessment if they met the inclusion criteria stated above and were reported for at least three of the included male or female patients. Each of these proteins/classes are listed below. 

### 3.1. α-1 Globulin

α-1 globulin was reported in 8 of the included females (6 increased, 2 no change) and 7 for the males (7 increased). Of the α-1 globulins in circulation, α-1 antitrypsin is thought to be the most abundant [27]. The main function is negative regulation of endopeptidase/proteolysis activity. Other α-1 globulins include α-1-glycoprotein, and α-1-HS glycoprotein. α1-fetoprotein is also a member of this globulin class, and this protein is the fetal analog of albumin with strong similarity in ligand binding.

### 3.2. α-2 Globulin

α-2 globulin was reported in 8 of the selected females (8 increased) and 6 males (6 increased). The most abundant α-2 globulins include macroglobulin and haptoglobin [27]. α-2 macroglobulin is known to inhibit coagulation and proteolysis, and haptoglobin is responsible for the recycling of heme iron and acting as an antioxidant. The α-2 globulin class also includes ceruloplasmin (which can bind copper), as well as retinol binding protein (RBP) and α-2 HS glycoprotein (each of which can bind FFA). 

### 3.3. β Globulin

β globulin was reported in 5 of the selected studies for females (4 increased, 1 no change) and 6 for males (6 increased). Transferrin is the most abundant β globulin, and it functions in iron ion transport [27]. It also plays a role in the immune system with positive regulation of B cell proliferation. Complement factors C3, C4, and C5 are also considered to be β globulins [24].

### 3.4. γ Globulin

γ globulin was reported for 8 of the selected females (3 increased, 5 no change) and 7 for the males (3 increased, 1 decreased, 3 no change). Immunoglobulins are the predominant γ globulins. The most abundant immunoglobulin found in circulation is IgG [27]. Other gamma globulins that are found in circulation include IgM, IgA, IgD, and IgE. 

### 3.5. α-1 Antitrypsin

α-1 Antitrypsin was reported 7 times in females (6 increased, 1 no change) and 3 times in males (3 increased). As mentioned, α-1 antitrypsin is the most abundant α-1 globulin, so this result is consistent with the result described above for its globulin class. Produced in the liver, this protein has anti-proteolytic activity and protects the lungs from damage through inhibiting neutrophil elastase activity [28].

### 3.6. Ceruloplasmin

Ceruloplasmin was reported in 3 of the selected females (1 increased, 2 no change) and 7 for the males (6 increased, 1 no change). The biological processes include acting as a ferroxidase and the transport of iron and copper [29].

### 3.7. Haptoglobin

Haptoglobin was reported in 4 of the selected females (2 increased, 2 no change) and 4 for males (2 increased, 2 no change). The main function of haptoglobin includes binding hemoglobin to assist in preventing renal and vascular injury [30]. 

### 3.8. Transferrin

Transferrin was measured in females 8 times (6 increased, 2 no change) and males 8 times (8 increased). Transferrin functions in iron transport, and it is synthesized by hepatocytes primarily [31]. 

### 3.9. Immunoglobulin M

Immunoglobulin M (IgM) was reported in 7 of the selected females (6 increased, 1 no change) and 4 in the males (1 increased, 3 no change). As a γ globulin, IgM plays a role in the immune system as immunomodulatory and protects against inflammation [32]. This protein is one of the first isotypes to develop in the immune system [32].

### 3.10. Immunoglobulin A

Immunoglobulin A (IgA) was reported in 7 of the selected females (7 no change) and 7 in males (2 increased, 5 no change). Functioning in the immune system, IgA neutralizes toxins and microbes, and it is the least abundant antibody in serum [33].

### 3.11. Immunoglobulin G

Immunoglobulin G (IgG) was reported in 7 of the selected females (3 increased, 4 no change) and 7 in the males (4 increased, 3 no change). IgG is the most abundant γ globulin in circulation, accounting for 10–20% of the protein in plasma [34]. It is the second-most abundance protein in plasma in healthy individuals, only inferior in abundance to albumin, and it plays a role in the immune response to antigens [34].

### 3.12. Fibrinogen

Fibrinogen was reported in 3 of the selected females (3 increased) and 2 for males (2 increased). Fibrinogen is the inactive precursor to fibrin; when converted to fibrin, it then activates blood coagulation and clot formation [35]. 

### 3.13. Apolipoprotein B

ApoB was measured in 2 of the selected females (2 increased) and 6 for males (5 increased, 1 no change). The main function is lipoprotein transport [36].

### 3.14. Transthyretin

Transthyretin was measured 5 times in the selected females (3 increased, 2 no change) and 1 time in the males (1 no change). Transthyretin functions as a transport protein for thyroxine and binds to RBP [37]. 

## 4. Discussion

Through this systematic review of published CAA case reports, we have assessed the response of the plasma protein profile to albumin deficiency in humans. It appears that any elevated expression of other proteins is rarely sufficient to maintain plasma protein abundance in a normal range. However, the upregulated expression of specific proteins and protein classes might still be of physiological relevance. Based upon a review of the literature, we determined that some protein classes were upregulated in CAA patients, even with greater that 50% of reported male or female patients exhibiting values outside the reference range. These proteins/classes of particular interest were α-1 globulin, α-2 globulin, β globulin, α-1 antitrypsin, ceruloplasmin, transferrin, IgM, fibrinogen, and apoB. The relative magnitude of these changes overall were modest though, and therefore it is important to be cautious in one’s interpretation of the observations. We conclude that the plasma protein abundances, contrary to some previous speculation, only change modestly in CAA. However, even though the changes are of limited magnitude, they may be supportive of physiological functions under the challenge of analbuminemia. The implications of these observations are discussed below. 

One may wonder how CAA patients can survive in the absence of albumin. Albumin is the most abundant protein in plasma with numerous important functions (transport of FFA, transport of various other ions, heme recycling, control of body fluid distribution, etc.). Thus, one may naturally assume that it is essential for survival. It is noteworthy that other medical conditions can lead to severe hypoalbuminemia (e.g., advanced kidney disease or liver disease), yet treating the albumin deficiency through albumin infusion typically leads to little or no measurable benefit [38,39,40]. In the literature on CAA, it has been suggested numerous times that total plasma protein concentration is defended by a marked increase in expression of other proteins [1,6,7]; this assertion has been used to rationalize how CAA patients can survive without albumin. However, based on our systematic review of the literature, it appears that this may not be the case, as CAA patient data included in this review consistently indicated low total protein concentration in plasma. Rather than a drastic remodeling of the plasma protein expression pattern, the absence of albumin appears to simply lead to a substantial reduction of the total plasma protein concentration (Figure 2), with the upregulation of other proteins being present but of limited magnitude (Table 2). Nonetheless, while the changes in plasma proteins in CAA do not fully defend the total protein concentration in plasma, still they may carry physiological relevance. 

While major protein classes have been measured, a global proteomics analysis has not been reported on CAA patients; therefore, it could be valuable to look to the basic science literature regarding the possible changes in low abundance proteins that could occur when albumin expression is absent. We recently assessed the plasma proteome through a mass spectrometry-based global proteomics analysis in albumin-deficient mice, and these results indicate that some minor changes occurred in the plasma proteome. Some of the proteins noted in the present review for CAA patients were also upregulated in albumin-deficient mice (apoB, transferrin, RBP, and α-2-HS-glycoprotein) [8]. It is difficult to compare analyses of globulin classes with mass spectrometry-based proteomics analysis, and the literature on CAA patients is largely based upon globulin class assessment. Nonetheless, there appears to be some alignment between human data on globulin classes with results from our previous analysis of the plasma proteome of albumin deficient mice. This albumin knockout mouse model exhibited elevation of transferrin, in agreement with both the transferrin findings in human CAA patients as well as the β globulin data in CAA patients, as β globulins include transferrin. This albumin knockout mouse also exhibited elevation of RBP and α-2-HS-glycoprotein, in agreement with an elevation of α-2 globulin in CAA patients, as α-2 globulins include these two proteins. Finally, as discussed further below, it also appears that both CAA patients and albumin deficient mice exhibit a moderate elevation of apoB expression. 

With the decrease of albumin in CAA patients, FFA transport between tissues may be slowed. In the articles selected for this systematic review, FFA concentrations were not reported. However, one might expect reduced plasma FFA concentration in humans with CAA, as analbuminemic mice (albumin knockout mice) exhibit reduced plasma FFA [8]. Studying the metabolic effects of decreased FFA in patients would require strict nutritional control, which does not usually occur in case studies. In our study of albumin knockout mice, we concluded that the altered lipid metabolism was likely a result of the fact that albumin is a circulating fatty acid-binding protein, and thus FFA trafficking was altered. In contrast, remodeling of the plasma proteome was considered as only a minor factor in the metabolic phenotype of the mouse model. With the decrease of FFA in plasma, hepatic steatosis was also reduced, and insulin sensitivity was improved [8]. Insulin sensitivity is not yet characterized in CAA patients. 

Though plasma long chain FFA are reduced in albumin KO mice, it is noteworthy that some meaningful level of this hydrophobic metabolite class still circulates [8]. In analbuminemic laboratory animals and humans with CAA, ApoB might be responsible for transporting a small amount of FFA to partially compensate for the lack of albumin. It is also noteworthy that RBP and α-2-HS-glycoprotein are upregulated in albumin deficiency [8] and each are known to bind FFA [41,42,43,44]. It appears that the changes in plasma protein expression patterns are geared toward compensating for lack of albumin’s FFA transport function. Secondary functions of albumin include transport of copper, heme iron, and thyroid hormones. Therefore, compensation for loss of this function in CAA may be indicated by upregulated ceruloplasmin (copper transport), haptoglobin and transferrin (iron transport and heme recycling), and transthyretin (thyroid hormone transport). To prevent oxidation and damage from occurring, iron is bound to transferrin for circulation. Therefore, there is little iron in its free form in circulation. Due to the increase of transferrin in the absence of albumin, it may be that transferrin is compensating for this role in albumin as well. Further, the α-2 globulin class is elevated in CAA patients, and this class of proteins includes haptoglobin and transferrin, further supporting the findings from the direct measurement of these proteins. Albumin is also known to bind some commonly prescribed drugs [45], and this binding could be expected to prolong the drugs’ half-lives and to improve the solubility of hydrophobic drugs. It is unknown if the remodeling of the plasma protein profile in CAA somehow alters the pharmacokinetics of drugs when albumin is absent. It is important to note that the changes in expression levels of these proteins in CAA are overall modest, yet they were still observed consistently in CAA patients. It is possible that these alterations in protein expression play some role in the ability of CAA patients to achieve a reasonable level of health despite lacking the most abundant plasma protein. Currently the molecular mechanism for upregulated expression of some proteins in analbuminemia is unknown. There may be feedback on gene expression, or the control may be held at the ribosomal translation level. Future work on tissues in animal models could address this question. 

In addition to albumin’s transport functions, as the most abundant protein in circulation, it plays a role in fluid balance. One of the most common, and usually the primary, symptom experienced by CAA patients is edema. This is likely due to impaired fluid balance control resulting from reduced total plasma protein concentration (Figure 2). In both humans with CAA (present report) and albumin knockout mice [8], the plasma protein expression pattern changes are not sufficient for achieving maintenance of normal total plasma protein concentration. Next, it is important to address the issue of body composition in CAA. In several of the articles reviewed, clinicians used the term “lipodystrophy” to describe patient complaints about fat distribution and body composition, typically in the hips and thighs with difficulty losing weight [2,11,14,15]. Most of these observations of high adiposity described a gynoid fat distribution, which is common in females but can occur in either sex. It may have been more accurate to use the term “overweight” or “obesity” to classify the observations, rather than the term “lipodystrophy.” Within the context of an ongoing obesity epidemic, it is important to keep in mind that many individuals are unhappy with their body weight and find it difficult to lose weight. True lipodystrophy may lead to severe ectopic lipid deposition and, therefore, fatty liver disease, but there was no mention of liver disease in the articles. While responses of mice and humans to a metabolic challenge are not always identical, it is worth noting that albumin knockout mice have normal body weight and low lipid storage in the liver, thus not suffering from a lipodystrophy phenotype [8]. Thus, while edema is well-accepted currently as the primary symptom of CAA, it is not currently established if CAA patients actually suffer from a heightened obesity predisposition or any lipodystrophy. This is why we did not use obesity as either an inclusion or exclusion criterion when selecting papers for the present analysis.

While it is accepted that albumin has important physiological roles, it simply cannot be ignored that the albumin deficiency in CAA patients has been reasonably well-tolerated in numerous instances. Thus, there may be impacts of albumin deficiency that ultimately exert some benefits that partially balance out the detriments. For example, it has been shown that albumin deficiency poses benefits in the setting of kidney disease. Kidney disease is characterized by oxidative stress and inflammation, and in those with kidney disease, the presence of albumin allows for the progression of the disease to occur more quickly. Kidney disease progression is actually slower in albumin knockout mice than wild-type mice [46]. Albuminuria is commonly noted in severe renal disease, and in such cases albumin may promote apoptosis of the tubular epithelial cells, leading to the progressive worsening of the pathology [47]. While it is generally believed that albumin has antioxidant and antiinflammatory roles in the body, it is also true that it can propagate the detriments of oxidative stress through the effects of chemically modified albumin upon cells. When modified through oxidative stress and glycation, the damaged form of albumin (e.g., glycated albumin) can cause cellular dysfunction in numerous cell types [48]. Furthermore, while insulin resistance is known to substantially increase the incidence of various diseases, albumin deficiency in mice leads to improved insulin sensitivity and reduced blood glucose levels [8]; the mechanisms appear to include reduced hepatic steatosis, increased adiponectin, and increased glucose transporter expression in adipose tissue, and other adaptations at the gene expression level in adipose tissue that would promote sequestration of FFA into neutral lipid stores [8]. Understanding the mechanisms for tolerance of analbuminemia in CAA patients requires that we acknowledge the multifactorial role of albumin in physiology, with a combination of detrimental effects of analbuminemia which may be partially balanced by some other impacts of albumin’s absence. 

Limitations. While the findings of this systematic review are informative, we note some limitations. In each case report, the patient values were compared to the reference ranges reported within that publication. As reference ranges represent the norms at a given time for a geographic location, those ranges can vary between locations and across time. While we felt it was most appropriate to compare the patient values to these reported reference ranges, we also acknowledge that this practice may have imparted some additional variance into the analysis results. We also note that each clinical laboratory may measure a protein class or an individual protein by methods that are different from other clinical laboratories; it is possible that this variability may have impacted the consistency of findings between studies. The sample size in the review also represents a fundamental limitation. There are multiple reasons why the literature on the topic of CAA is limited. As the phenotype of CAA is often not notable, it is likely that there are many undiagnosed cases of CAA; this would be particularly common in cultures in which routine preventive care is not a common practice, or in which preventative medicine practices do not include biochemical assessment of serum albumin in apparently healthy individuals. Another reason for the limited literature would be that only a subset of patients with any medical condition are described in a published case report, thus making their diagnosis unavailable to literature searches. Nonetheless, we were able to identify a sufficient number of case reports to compile the first-ever systemic review of plasma proteins in the CAA patient population, and we expect these findings to be impactful to the fields of clinical investigation and metabolic regulation. 

## 5. Conclusions 

In conclusion, a systematic review of the literature indicated that the remodeling of the plasma protein distribution occurs consistently in CAA patients, but the magnitude of the changes are modest. Some protein expression levels are consistently above the reference range, yet the fold changes are limited; in most patients fold changes are within approximately two-fold of normal for various proteins or protein classes. The physiological impacts of albumin deficiency are complex, manifesting as a combination of the effects of a low total protein concentration as well as disruption of albumin’s metabolic functions. Yet remarkably, humans can survive and function without albumin, even in the absence of drastic changes in the expression of other proteins. The upregulated expression of specific proteins in CAA patients may have their impacts limited by the modest magnitude of the changes. However, it is also possible that these limited changes are in fact needed for health or even survival in analbuminemia. The plasma proteins identified by this systematic review may be relevant to other clinical conditions that lead to hypoalbuminemia and analbuminemia. Continued research is needed to further characterize metabolic control in states of analbuminemia and hypoalbuminemia. 

## Figures and Tables

**Table 1:** **PICOS**. Table describing population, intervention, comparison, outcome, and setting for the systematic review. The research question is also specified.

Parameter	Description
Population	Males and females diagnosed with CAA which is caused by a mutation in the albumin gene
Intervention	No intervention is required for inclusion
Comparison	Clinical reference ranges
Outcome	Biochemical assay results from analysis of plasma or serum
Setting	Case reports of albumin deficiency reasonably attributable to homozygous albumin gene mutation.
Research Question	How does the plasma protein expression pattern respond to the lack of albumin in CAA patients?

**Table 2:** **Fold change of proteins analyzed.** If a protein concentration was above the reference range reported by the authors, fold change was calculated by dividing the value by the upper level of the reference range. If a protein concentration was below the reference range reported by the authors, fold change was calculated by dividing the value by the lower level of the reference range. Fold change above 1 indicates expression above the reference range, and below 1 indicates expression level below the reference range. NS, not significant (expression level was reported but was within the stated reference range).

Paper	Sex	α-1 Globulin	α-2 Globulin	β Globulin	γ Globulin	α-1 Antitrypsin	Ceruloplasmin	Haptoglobin	Transferrin	IgM	IgA	IgG	Fibrinogen	ApoB	Transthyretin
Berglund et al., 2015 [11]	F	NS	2.3	1.3	1.1	Not reported	Not reported	Not reported	2.0	2.4	NS	NS	1.2	Not reported	Not reported
Caridi et al., 2019 [12]	F (patient 2)	1.5	1.3	Not reported	NS	1.2	NS	1.1	1.6	Not reported	Not reported	Not reported	Not reported	Not reported	Not reported
Caridi et al., 2018 [13]	F (patient 1)	NS	1.1	1.03	1.1	NS	Not reported	1.3	1.7	NS	NS	1.2	Not reported	Not reported	Not reported
	F (patient 2)	1.2	1.5	Not reported	NS	2.0	Not reported	Not reported	NS	1.5	NS	1.1	Not reported	Not reported	1.3
	F (patient 3)	1.3	1.5	Not reported	NS	2.1	Not reported	Not reported	NS	1.2	NS	NS	Not reported	Not reported	NS
Caridi et al., 2012 [3]	F	Not reported	Not reported	Not reported	Not reported	Not reported	Not reported	Not reported	Not reported	1.1	NS	1.04	Not reported	Not reported	Not reported
Dagnino et al., 2010 [14]	F	1.2	1.2	NS	1.1	Not reported	Not reported	Not reported	Not reported	Not reported	Not reported	Not reported	Not reported	Not reported	Not reported
Dammacco et al., 1980 [4]	F	Not reported	Not reported	Not reported	Not reported	1.8	NS	NS	1.4	1.7	NS	NS	1.3	Not reported	1.6
Koot et al., 2004 [2]	F	1.3	2.0	1.7	NS	1.6	1.4	NS	2.3	1.3	NS	NS	1.4	1.1	NS
Newstead et al., 2004 [15]	F	1.8	1.6	1.5	NS	Not reported	Not reported	Not reported	Not reported	Not reported	Not reported	Not reported	Not reported	Not reported	Not reported
Suppressa et al., 2019 [16]	F	Not reported	Not reported	Not reported	Not reported	1.3	Not reported	Not reported	1.7	Not reported	Not reported	Not reported	Not reported	1.1	1.2
Bibi et al., 2012 [17]	M (patient 1)	2.0	1.1	1.1	0.7	Not reported	Not reported	Not reported	Not reported	Not reported	Not reported	Not reported	Not reported	Not reported	Not reported
Campagnoli et al., 2008 [18]	M	Not reported	Not reported	Not reported	Not reported	1.8	1.7	1.4	2.3	1.1	NS	NS	Not reported	Not reported	Not reported
Campagnoli et al., 2006 [19]	M (patient 1)	Not reported	Not reported	Not reported	Not reported	Not reported	2.5	Not reported	1.6	Not reported	1.3	1.3	Not reported	1.4	Not reported
	M (patient 2)	Not reported	Not reported	Not reported	Not reported	Not reported	1.6	Not reported	1.6	Not reported	NS	1.1	Not reported	1.6	Not reported
	M (patient 3)	Not reported	Not reported	Not reported	Not reported	Not reported	1.5	Not reported	1.3	Not reported	NS	1.02	Not reported	1.1	Not reported
Caridi et al., 2019 [12]	M (patient 1)	1.3	NS	Not reported	1.7	1.3	1.4	NS	1.2	Not reported	Not reported	Not reported	Not reported	Not reported	Not reported
Caridi et al., 2016 [20]	M	2.4	1.6	1.4	NS	Not reported	Not reported	Not reported	1.4	NS	NS	NS	Not reported	Not reported	Not reported
Caridi et al., 2013 [21]	M (patient 2)	1.1	1.2	1.2	1.1	Not reported	Not reported	Not reported	1.4	Not reported	Not reported	Not reported	1.2	Not reported	Not reported
Caridi et al., 2012 [7]	M	1.2	2.5	1.9	NS	Not reported	Not reported	Not reported	Not reported	Not reported	Not reported	Not reported	Not reported	2.1	Not reported
Caridi et al., 2008 [5]	M	2.0	2.5	1.6	NS	Not reported	NS	Not reported	Not reported	NS	1.03	NS	1.5	NS	Not reported
Dagnino et al., 2011 [22]	M	1.7	1.7	1.3	2.2	Not reported	Not reported	Not reported	Not reported	Not reported	Not reported	Not reported	Not reported	Not reported	Not reported
Weigand et al., 1983 [23]	M	Not reported	Not reported	Not reported	Not reported	2.3	2.4	2.8	1.2	NS	NS	1.03	Not reported	1.3	NS

## Data Availability

Data will be made available upon reasonable request.
